# Genetically Engineered Live-Attenuated Middle East Respiratory Syndrome Coronavirus Viruses Confer Full Protection against Lethal Infection

**DOI:** 10.1128/mBio.00103-21

**Published:** 2021-03-02

**Authors:** Javier Gutiérrez-Álvarez, José M. Honrubia, Raúl Fernández-Delgado, Li Wang, Carlos Castaño-Rodríguez, Sonia Zúñiga, Isabel Sola, Luis Enjuanes

**Affiliations:** a Department of Molecular and Cell Biology, Centro Nacional de Biotecnología (CNB-CSIC), Madrid, Spain; Vanderbilt University Medical Center

**Keywords:** genome engineering, MERS-CoV, coronavirus, vaccines

## Abstract

There are no approved vaccines against the life-threatening Middle East respiratory syndrome coronavirus (MERS-CoV). Attenuated vaccines have proven their potential to induce strong and long-lasting immune responses. We have previously described that severe acute respiratory syndrome coronavirus (SARS-CoV) envelope (E) protein is a virulence factor. Based on this knowledge, a collection of mutants carrying partial deletions spanning the C-terminal domain of the E protein (rMERS-CoV-E*) has been generated using a reverse genetics system. One of these mutants, MERS-CoV-E*Δ2in, was attenuated and provided full protection in a challenge with virulent MERS-CoV after a single immunization dose. The MERS-CoV-E*Δ2in mutant was stable as it maintained its attenuation after 16 passages in cell cultures and has been selected as a promising vaccine candidate.

## Abstract (precis)

The emergence of the new highly pathogenic human coronavirus SARS-CoV-2 that has already infected more than 80 million persons, killing nearly two million of them, clearly indicates the need to design efficient and safe vaccines protecting from these coronaviruses. Modern vaccines can be derived from virus-host interaction research directed to the identification of signaling pathways essential for virus replication and for virus-induced pathogenesis, in order to learn how to attenuate these viruses and design vaccines.

## INTRODUCTION

Middle East respiratory syndrome coronavirus (MERS-CoV) is a zoonotic emergent coronavirus (CoV) from dromedary camels that appeared in the human population in 2012 (1). Since then, it has been reported in 27 countries (https://www.who.int/emergencies/mers-cov/en/). The clinical signs caused by MERS-CoV in humans vary from asymptomatic to severe respiratory disease and death (2–4), and include symptoms like fever, shortness of breath, and, in critical cases, pneumonia resulting in mechanical ventilation and support in an intensive care unit (5–7). Most of the MERS-CoV contagions occur in hospital settings, limiting the transmissibility. However, the risk of restricted or global epidemics is considerable, as has been demonstrated by periodic transmission outside the Middle East, specifically by the outbreak in 2015 in South Korea, which resulted in 186 cases and 38 deaths derived from a single traveler (8–11). There are no specific treatments or vaccines against MERS-CoV approved for use in humans yet (12). Nonetheless, with the emergence and the high impact of SARS-CoV-2 (13), several tested compounds have been found to be effective against CoVs, both *in vitro* and in patients (14–25).

Coronaviruses belong to the *Nidovirales* order and have positive-sense RNA genomes that range in size between 26 and 32 kb (26–28). CoVs infect animals and humans. Seven human CoVs have been identified, of which four CoVs, 229E, NL63, OC43, and HKU1, cause mild to moderate infections (29), whereas the other three human CoVs, SARS-CoV, MERS-CoV, and SARS-CoV-2, are virulent and have caused deadly outbreaks during the past 2 decades (30–34).

SARS-CoV caused the first deadly human CoV outbreak in 2003, which was contained in 6 months (35, 36). The SARS-CoV outbreak resulted in around 8,000 laboratory-confirmed infections worldwide with 774 deaths and a case-fatality rate of 9.6% (37). In 2012, the MERS-CoV was identified as the causative agent of MERS in Saudi Arabia (38, 39). The MERS-CoV outbreak of 2012 caused a case-fatality rate of 34.4% from 2,519 laboratory-confirmed cases and 866 associated deaths (40). Fortunately, the transmission efficacy between individuals is highly limited and restricted to special settings. Then, at the end of 2019, SARS-CoV-2 was responsible for another outbreak in Wuhan, China (41–43). As of December 2020, close to 80 million confirmed cases of SARS-CoV-2 infections with around 2.2% resulting deaths were reported worldwide, leading to the largest pandemic in the present century (44).

SARS- and MERS-CoV cause a severe disease, even in immunocompetent, healthy individuals (33). Patients infected with SARS-CoV present with symptoms resembling atypical pneumonia, exhibiting fever, chills, headache, malaise, myalgia, and dry cough (45–47). Those infected with MERS-CoV report similar nonspecific symptoms but demonstrate a much higher case-fatality rate, particularly for elderly persons and those with underlying medical conditions (48, 49). In some cases, a small proportion of both SARS and MERS patients develop gastrointestinal symptoms such as nausea, vomiting, or diarrhea (32). All these facts raise the need for the development of efficient vaccines for these human CoVs.

An ideal vaccine for MERS-CoV should induce potent humoral and cellular responses without leading to immunization side effects. Likewise, it must be able to induce a good immune response in mucosa. Inactivated SARS-CoV and MERS-CoV vaccines may cause eosinophilia in the lung of immunized mice (50–53). In addition, vaccines developed against SARS-CoV that include complete spike (S) protein have been associated with other side effects, such as antibody-dependent enhancement of infectivity (54), which has raised serious concerns about the safety of vaccines based on the complete MERS-CoV S protein (55–59). In contrast, immunization with recombinant subunit vaccines, which exclusively include the S1 subunit or the MERS-CoV S protein receptor binding domain (RBD), prevents the occurrence of these side effects. However, these types of vaccines have the limitation of requiring more than one booster dose to achieve long-lasting protection (60–64).

Currently, the most promising strategies for developing effective vaccines are based on DNA vaccines, vectored vaccines, and reverse genetics-engineered live-attenuated vaccines (LAV). In all three cases, the vaccine acts as an antigen carrier that converts the cell that incorporates it into an antigen production factory. Indeed, one DNA and two vectored vaccines against MERS-CoV have advanced into human trials (65). LAV induce highly potent and balanced cellular and humoral responses since the route of antigen presentation is similar to that of natural infection. Often these vaccines confer long-lasting immunity. However, they may have a drawback related to the safety of the vaccine, due to the possible reversion of the vaccine virus to virulence, and the limitation that it cannot be administered to immunocompromised individuals (66).

The study of the molecular mechanisms of virus pathogenesis allows the identification of viral factors involved in virulence and virus-host interaction (67). Using reverse genetics systems, several virulence factors can be eliminated to obtain live-attenuated viruses in which the composition and genetic stability are precisely known (68–72), thus minimizing the risk of reversion to a virulent phenotype (73). Furthermore, these vaccines can be easily designed and prepared in a relatively short period of time as a quick response to outbreaks or epidemics.

Our group has described that the Envelope (E) protein of SARS-CoV is implicated in virulence through the interaction with several cellular proteins (69, 74, 75). These interactions have been studied in the context of SARS-CoV infection, and it is unknown whether they are reproduced by E proteins from other CoVs. The study of these interactions in CoVs such as MERS-CoV could help to identify potential targets for the development and application of effective treatments against infections caused by these viruses.

In this study, we describe the construction of a collection of mutants carrying partial deletions within the C-terminal domain of the MERS-CoV E protein. Among them, one mutant was attenuated and conferred protection with a single dose in a lethal MERS-CoV challenge. The attenuation of this mutant seemed related to the absence of one predicted linear motif: a ligand of forkhead-associated (FHA) domain.

## RESULTS

### Viability and selection of MERS-CoV-E* mutants.

**FIG 1:**
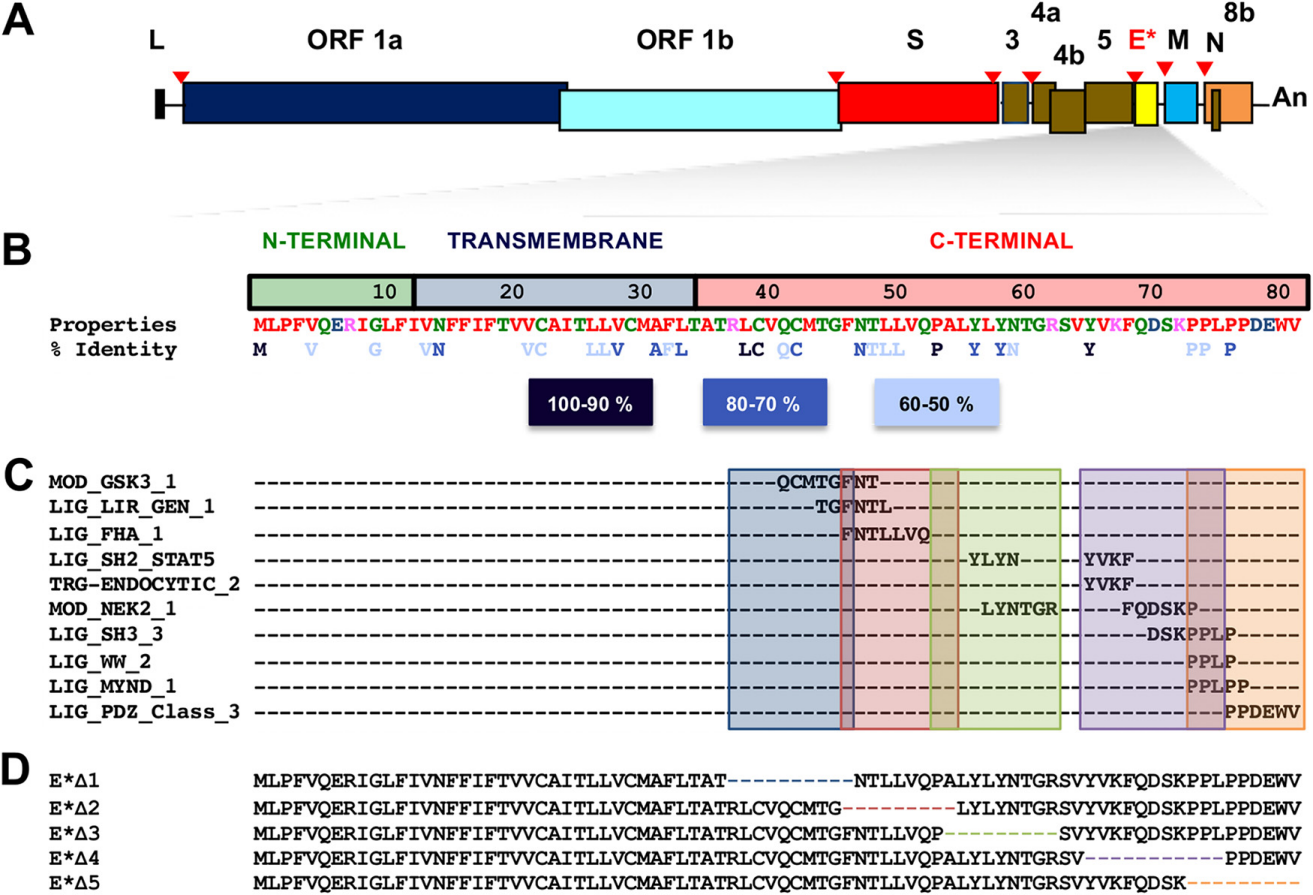
MERS-CoV mutants with partial deletions in the C-terminal domain of protein E. (A) Schematic of the MERS-CoV genome. L, leader sequence; An, poly(A) tail. (B) The structural domains of MERS-CoV E protein and amino acid positions are indicated. The color code of amino acid properties is shown. Red, small and hydrophobic (includes aromatics except tyrosine). Blue, acidic. Magenta, basic (except histidine). Green, with hydroxyl, thiol, and amine groups (and also including glycine). The color code of amino acid percent identity is indicated at the bottom of the figure. (C) Location of the functional motifs predicted in protein E. Note that LIG_LIR_GEN_1 is also named Atg8 binding motif. (D) Mutants with partial deletions of protein E (MERS-CoV-E*).

The C-terminal domain of the envelope protein (E) was analyzed with the Eukaryotic Linear Motif resource (http://elm.eu.org/) to identify potential functional motifs. In addition, a multiple sequence alignment of 10 different CoV E proteins was performed to localize several conserved residues (https://www.ebi.ac.uk/Tools/msa/clustalo/). Combining the results of the two analyses, five MERS-CoV mutants (MERS-CoV-E*) were designed to contain deletions of 9 to 11 amino acids spanning the C-terminal domain exposed to the cytosol (Fig. 1). Each of the five mutations deleted different predicted linear motifs and conserved positions (Fig. 1C and D; see also Table S1 in the supplemental material). One of these functional motifs was the PDZ binding motif (PBM), previously identified in our laboratory as a virulence factor in SARS-CoV infection (69). It has been demonstrated that these PBMs are highly relevant for the virulence of different viruses, like influenza virus (76) and rabies virus (77), among others (78). *In silico*, the engineered deletions or the substitutions did not generate new motifs in the resultant MERS-CoV-E* mutants (Table S1).

10.1128/mBio.00103-21.2 TABLE S1 Prediction of functional motifs of MERS-CoV E protein. Download Table S1, DOCX file, 0.02 MB. Copyright © 2021 Gutiérrez-Álvarez et al. 2021 Gutiérrez-Álvarez et al. https://creativecommons.org/licenses/by/4.0/ This is an open-access article distributed under the terms of the Creative Commons Attribution 4.0 International license.

**FIG 2:**
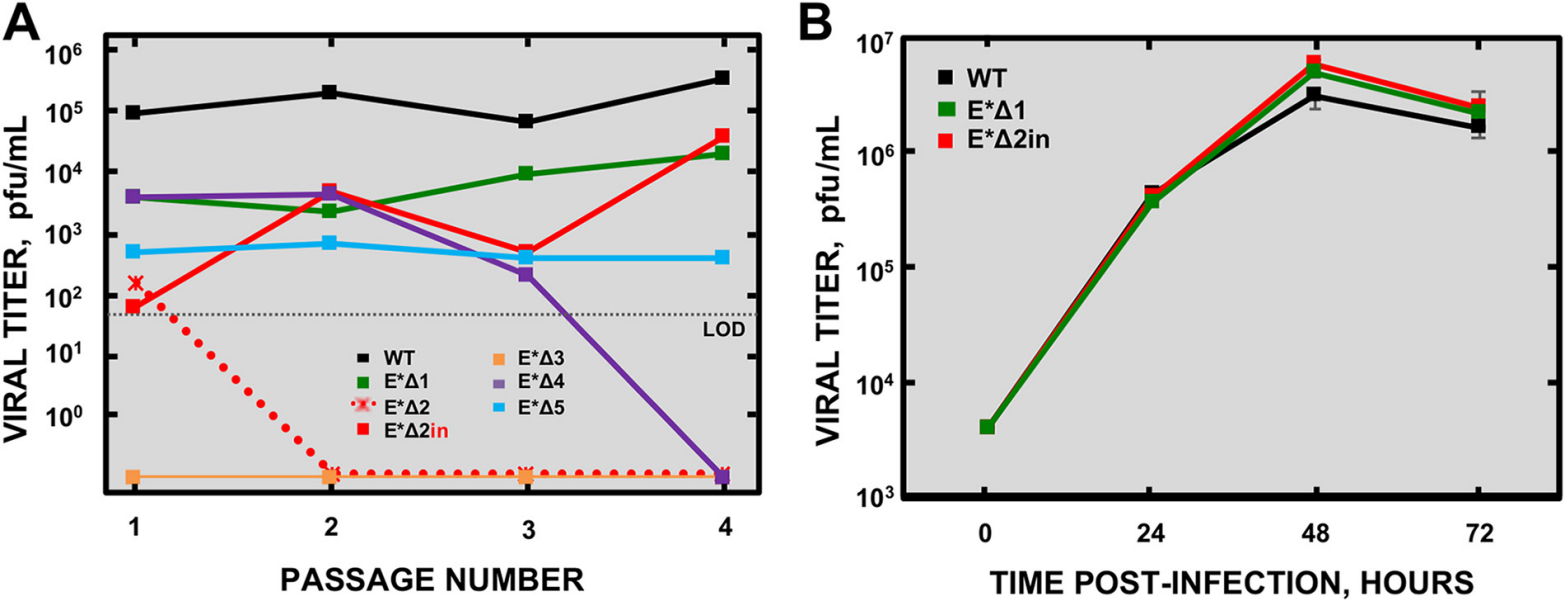
Growth of MERS-CoV-E* mutants in Huh-7 cells. (A) Titers of MERS-CoV-E* mutants after rescue during virus passage in cell culture. One-third of the volume of the supernatant was passaged (blind passages). Three independent cDNA clones were rescued for every mutant, but only one of them is represented in the figure. In the case of the MERS-CoV-E*Δ2 mutant, two clones were represented: one of the nonviable clones (E*Δ2) and the one that was viable and evolved a 15-nucleotide insertion (E*Δ2in). The MERS-CoV-E*Δ5 mutant was titrated by an immunofluorescence focus formation detection assay. (B) Growth kinetics of the MERS-CoV-E* mutants. A multiplicity of infection (MOI) of 0.001 was used for all selected mutants (MERS-CoV-E*Δ1 and E*Δ2in; the MERS-CoV-WT was used as a control). LOD, limit of detection.

**FIG 3:**
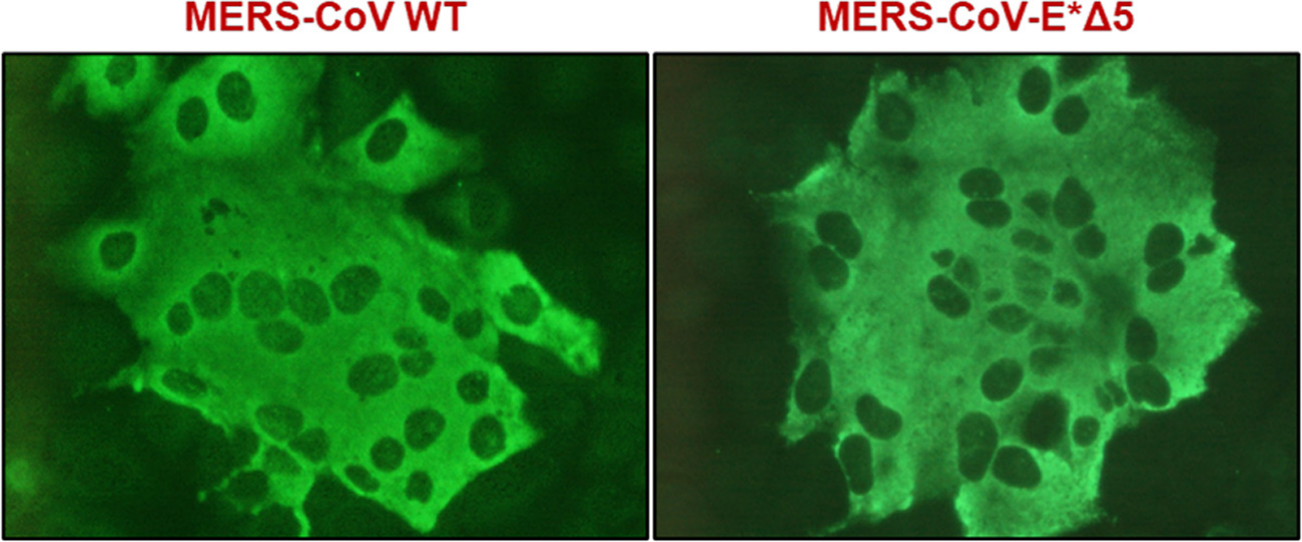
Comparison of the MERS-CoV-WT and MERS-CoV-E*Δ5 virus foci of infection. Huh-7 cells were infected with passage 4 virus after rescue. At 17 h postinfection, infection foci were revealed with a MERS-CoV N protein-specific antibody.

Three independent infectious clones (bacterial artificial chromosomes [BACs]) were rescued for each mutant: MERS-CoV-E*Δ1, E*Δ2, E*Δ3, E*Δ4, and E*Δ5. These viruses were passaged four times in Huh-7 cells every 72 h (Fig. 2A). The original sequence was compared with the sequence of the virus from passage 4. Virus titers and viability were analyzed during these passages. Among the five mutants, only four were rescued: MERS-CoV-E*Δ1, E*Δ2, E*Δ4, and E*Δ5. Despite the E*Δ1 mutant being viable, its titers were 1 logarithmic unit lower than MERS-CoV-WT (wild type). E*Δ1 lysis plaques were smaller than MERS-CoV-WT (data not shown). Although the E*Δ4 mutant was rescued, it was lost through the passages, indicating that it was not viable. In the case of the E*Δ5 mutant, no lysis plaques were detected in any of the passages, although genomic and subgenomic RNAs were present (data not shown). To confirm that the E*Δ5 mutant was, indeed, rescued and viable, the titer from passages 1 to 4 was analyzed by a focus-forming immunofluorescence assay. At 17 h postinfection (hpi), there were no differences in the size and shape of E*Δ5 foci compared to MERS-CoV-WT (Fig. 3). Nonetheless, the titer was 100-fold lower (Fig. 2A).

**FIG 4:**
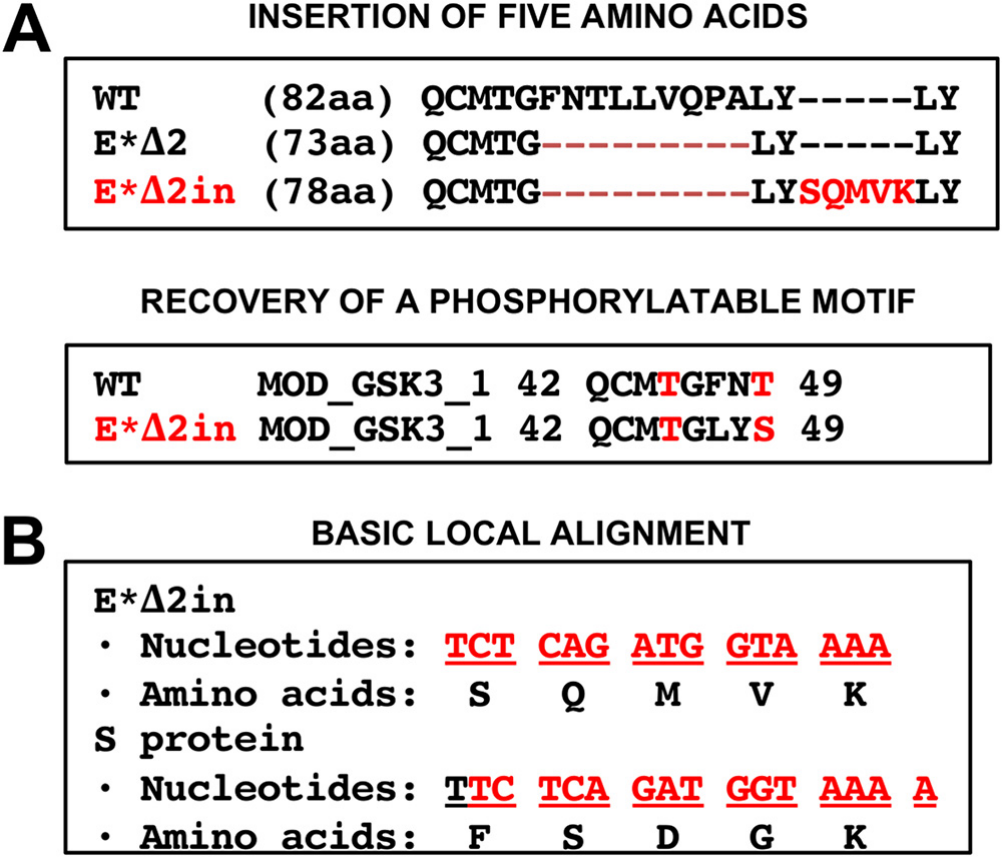
Description and origin of the E*Δ2in insertion. (A) Sequence of the MERS-CoV-E*Δ2in mutant with the insertion of five amino acids and recovered functional motifs. GSK3 phosphorylated motif recovered in the MERS-CoV-E*Δ2in: QCMTGLYS. Phosphorylatable residues are indicated in red. (B) Basic Local Alignment of the 15 nucleotides inserted within the E gene that led to the generation of the MERS-CoV-E*Δ2in mutant. The 15-nucleotide sequence is indicated in red, and below are the corresponding amino acids that are encoded within the S or the E*Δ2in frame sequences.

The E*Δ2 mutant was particularly interesting, since viable virus was rescued from just one of the three independent cDNA clones: E*Δ2 clone 2 (her called E*Δ2in) (Fig. 2A). The sequence of the virus at passage 4 was analyzed, and an insertion of 15 nucleotides (3′-TCTCAGATGGTAAAA-5′) was identified, encoding five amino acids (SQMVK) following the engineered original mutation (Fig. 4A). The E*Δ2 deletion eliminates three predicted functional motifs: (i) a GSK3 (glycogen synthase kinase 3) phosphorylation site; (ii) an Atg8 (autophagy-related protein) protein family ligand, also named LIG_LIR_GEN_1; and (iii) an FHA (forkhead-associated) phosphopeptide ligand (Table S1). The insertion of five amino acids identified in the MERS-CoV-E*Δ2in mutant after four passages restored a GSK3 phosphorylation site, but not the FHA phosphopeptide ligand or the Atg8 protein family ligand (Table S1).

In order to identify the origin of the introduced sequence, a Basic Local Alignment was performed with BLAST (https://blast.ncbi.nlm.nih.gov/Blast.cgi) using the 15-nucleotide sequence insertion (3′-TCTCAGATGGTAAAA-5′) against all the annotated MERS-CoV sequences. There was a 100% identity with a sequence of 15 nucleotides within the S gene, although the frame sequence was different from the E*Δ2in gene, rendering a different sequence of amino acids (Fig. 4B). Most likely, the nucleotide sequence incorporated into the E gene was derived from the S gene, restoring the GSK3 phosphorylation site and E protein activity and leading to a viable E*Δ2in mutant.

E*Δ4 and E*Δ5 mutants were discarded, due to the lack of viability or the low titer, respectively. On the other hand, plaques were isolated from passage 4 of E*Δ1 and E*Δ2in mutants for further characterizations. The growth kinetics of these mutants were analyzed in Huh-7 cells infected with a multiplicity of infection (MOI) of 0.001 (Fig. 2B). E*Δ1 and E*Δ2in mutants reached titers similar to MERS-CoV-WT.

### Evaluation *in vivo* of the attenuation of E*Δ1 and E*Δ2in mutants.

**FIG 5:**
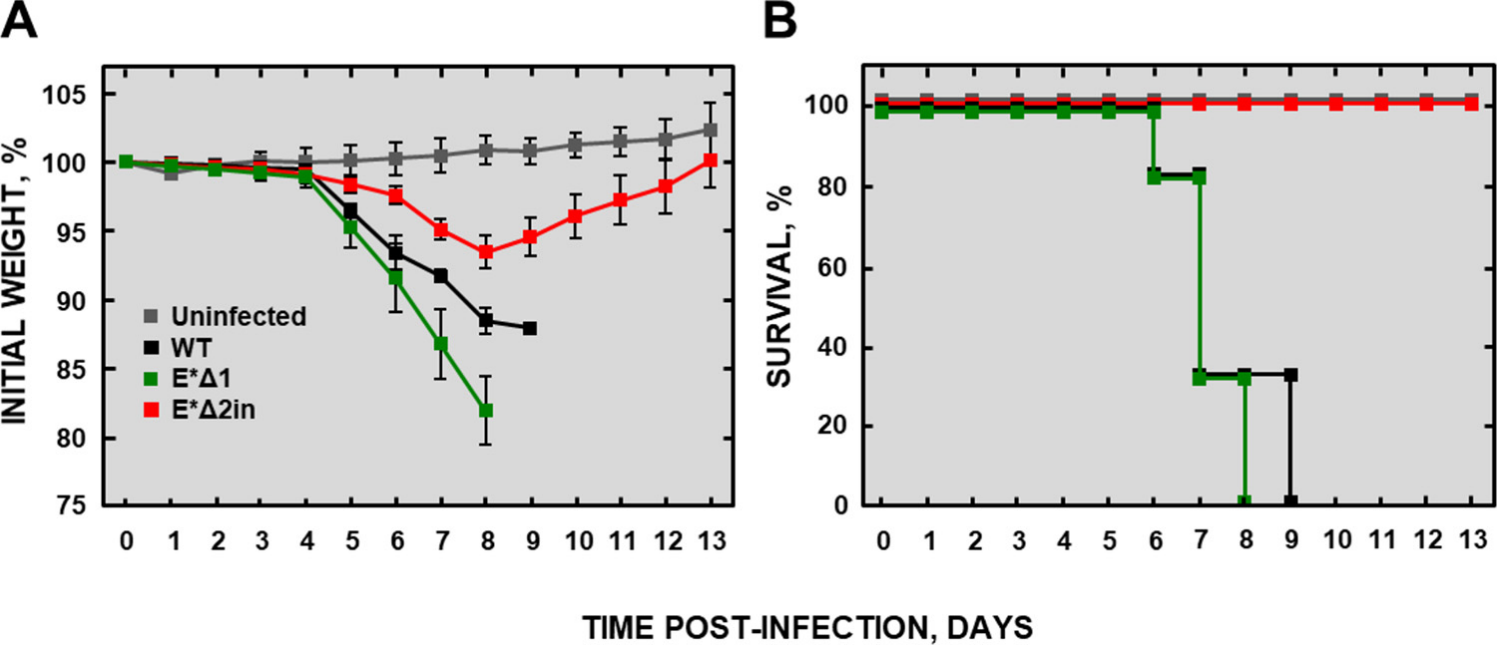
Evaluation of the attenuation of the MERS-CoV-E*Δ1 and MERS-CoV-E*Δ2in mutants in K18-hDPP4 mice. (A) Clinical signs are represented as weight losses of the infected mice. (B) Virulence and attenuation are represented as survival. Differences in weight loss are represented as the mean ± standard error of the mean.

The attenuation of the selected MERS-CoV-E* mutants was evaluated in transgenic K18-hDPP4 mice (79) (Fig. 5). Each animal was intranasally inoculated with 5 × 10^3^ PFU of each virus. As expected, the mice infected with MERS-CoV-WT lost weight rapidly, and all of them died between 6 and 9 days postinfection (dpi). Interestingly, the mice infected with the E*Δ1 mutant lost weight and died too, indicating that the E*Δ1 mutant was not attenuated, and therefore, it was discarded. In contrast, mice infected with the E*Δ2in mutant lost weight slightly, but all of them survived and recovered from the infection, indicating that this mutant was attenuated and a promising vaccine candidate.

### The attenuation of the E*Δ2in mutant is not associated with deficiencies in growth, replication, or transcription in the lungs.

**FIG 6:**
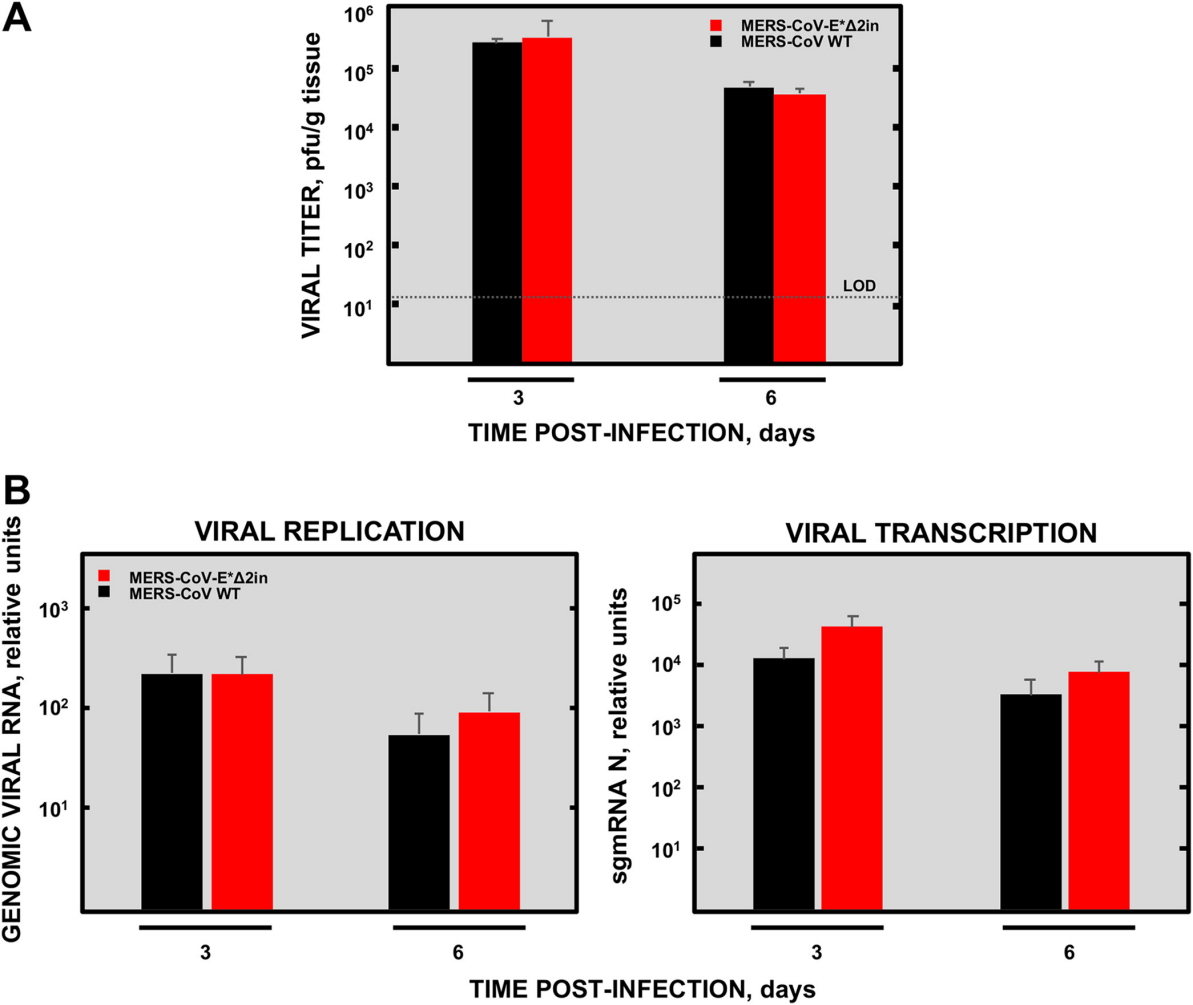
Growth, replication, and transcription of MERS-CoV-WT and MERS-CoV-E*Δ2in viruses in the lungs of infected mice. (A) Viral titers in the lung. (B) Replication and transcription levels. Results are expressed as the mean ± standard deviation. LOD, limit of detection.

The E*Δ2in mutant was selected as a vaccine candidate due to its capacity to reach titers similar to MERS-CoV-WT in cell cultures (Fig. 2B). For a better description of E*Δ2in mutant infection in the lung, mice were intranasally infected with 5 × 10^3^ PFU to study lung pathology, viral growth, and viral replication and transcription at 3 and 6 dpi. No significant differences in lung viral titer between the E*Δ2in mutant and MERS-CoV-WT were observed (Fig. 6A), similar to what was observed in cell cultures. Furthermore, no significant differences were found in either genomic or subgenomic viral RNAs levels between the E*Δ2in mutant and MERS-CoV-WT (Fig. 6B).

**FIG 7:**
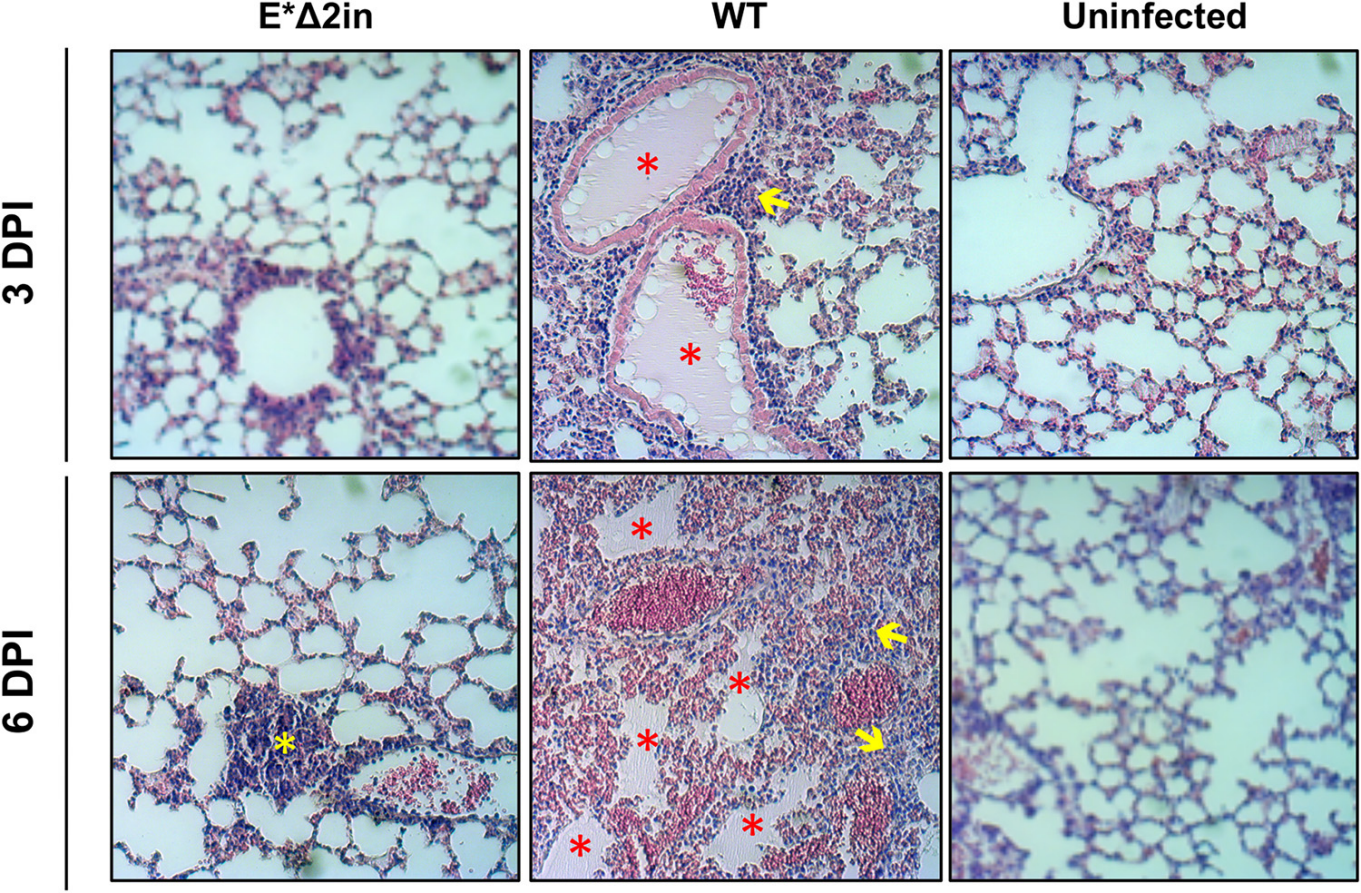
Histopathology induced by MERS-CoV-E*Δ2in (E*Δ2in) mutant in the lungs of infected mice. DPI, days postinfection. In the lungs of the mice infected with the MERS-CoV-WT (WT), areas with edema (red asterisks) and cell infiltrates (yellow arrows) are observed, at both 3 and 6 DPI. In the lungs of mice infected with MERS-CoV-E*Δ2in, some small perivascular and peribronchiolar infiltrates could be observed at 6 DPI (yellow asterisk).

During the histological examination, the appearance of lungs from mice infected with the E*Δ2in mutant at 3 dpi was similar to the lungs of the noninfected mice (Fig. 7). In contrast, the lungs of the mice infected with MERS-CoV-WT showed incipient infiltrates in several perivascular and peribronchiolar areas. At 6 dpi, the lungs of the mice infected with MERS-CoV-WT presented generalized infiltration and parenchyma consolidation, as well as edema in the airspaces, whereas the lungs of the mice infected with the E*Δ2in mutant were similar to those from noninfected mice. In general, the histopathology associated with E*Δ2in infection was significantly lower than that associated with MERS-CoV-WT infection, although some little and scattered infiltrates could be observed surrounding bronchioles and blood vessels at 6 dpi (Fig. 7). These results demonstrated that the survival of the mice infected with the E*Δ2in mutant was correlated with a mild or absent pulmonary infiltration, and its attenuation was not due to a decrease in viral titers, or a difference in viral replication and transcription in the lungs compared to MERS-CoV-WT.

### The E*Δ2in mutation might be associated with a reduced brain tropism.

Since K18-hDPP4 mice developed brain disease after infection with MERS-CoV (79), the presence of the virus was analyzed in this organ to test whether the attenuation of E*Δ2in was related to a reduction of the viral replication (Fig. S1). No virus was detected at 3 dpi in the brain of mice infected with MERS-CoV-WT or E*Δ2in, an observation consistent with previous data in which it was described that virus titers in the brain of K18-hDPP4 mice were low or null at short times after infection (79). Although at 6 dpi the wild-type virus showed relatively high titers (around 10^5^ PFU/g of tissue), the levels of virus in the deletion mutant E*Δ2in were reduced by 1,000-fold, illustrating the significant attenuation of this deletion mutant virus. The presence of both MERS-CoV-WT and E*Δ2in in the brain of the transgenic mice was, most likely, due to the artificial expression of large amounts of hDPP4 in this tissue (79). In addition, it must be considered that the potential growth of the virus in the brain of humans should not be promoted by the lower levels of hDPP4 in human brains (80), and by the administration of the vaccine candidate intramuscularly instead of intranasally, as done in this study. Nevertheless, these observations also indicate the need to introduce complementary safety measures in the vaccine candidate before it could be administered to humans.

10.1128/mBio.00103-21.1 FIG S1 Growth of MERS-CoV-WT and MERS-CoV-E*-Δ2in viruses in the brain of infected mice. Viral titers are indicated as the mean ± standard deviation. LOD: limit of detection. Download FIG S1, TIF file, 0.3 MB. Copyright © 2021 Gutiérrez-Álvarez et al. 2021 Gutiérrez-Álvarez et al. https://creativecommons.org/licenses/by/4.0/ This is an open-access article distributed under the terms of the Creative Commons Attribution 4.0 International license.

### Protection elicited by E*Δ2in mutant in a lethal challenge with MERS-CoV-WT.

**FIG 8:**
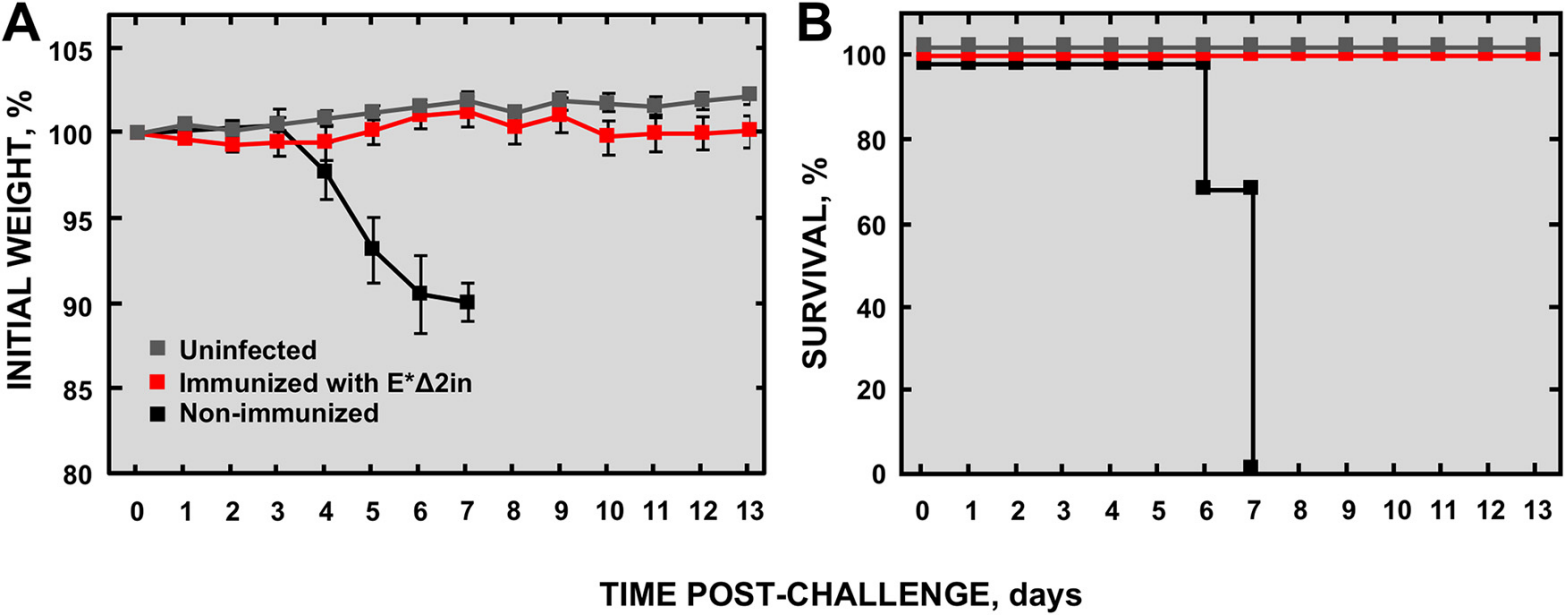
Protection conferred by attenuated MERS-CoV-E*Δ2in mutant in K18-hDPP4 mice. (A) Clinical signs are represented as weight losses of the challenged mice. (B) Survival of the mice immunized with the attenuated mutant E*Δ2in. Differences in weight loss are represented as the mean ± standard error of the mean.

Mice immunized with 5 × 10^3^ PFU of E*Δ2in were challenged with 5 × 10^3^ PFU of MERS-CoV-WT at 21 days postimmunization (dpim) (Fig. 8). Nonimmunized mice lost weight and died at 6 and 7 days postchallenge (dpc). However, the mice immunized with E*Δ2in vaccine candidate survived, and no weight loss was observed. These results demonstrated that a single immunization with 5 × 10^3^ PFU of E*Δ2in elicited full protection in a challenge with a lethal dose of MERS-CoV-WT.

### The E*Δ2in mutant elicited sterilizing immunity in a challenge with a high dose of MERS-CoV-WT.

Mice immunized with 5 × 10^3^ PFU of E*Δ2in mutant were challenged at 21 dpim with a high dose of MERS-CoV-WT (1 × 10^5^ PFU). Lung samples were taken at 2, 4, and 8 dpc to determine lung histopathology, viral titer, and levels of viral replication and transcription.

**FIG 9:**
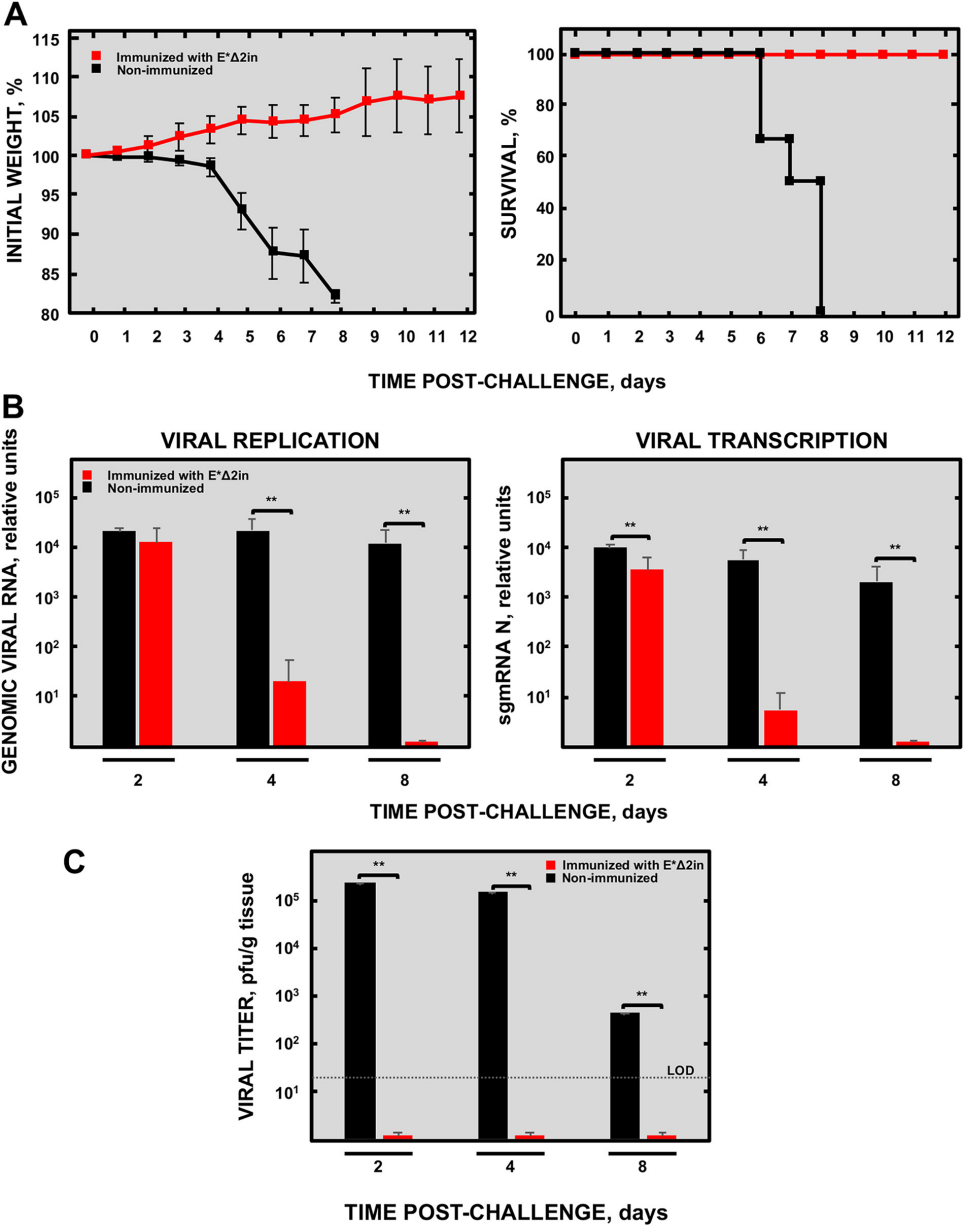
The challenge with a high dose of MERS-CoV-WT of mice immunized with the MERS-CoV-E*Δ2in mutant showed sterilizing immunity. (A) Weight loss (left) and survival (right). Differences in weight loss are represented as the mean ± standard error of the mean. (B) Viral replication (left) and transcription (right) in the lungs of challenged mice. (C) MERS-CoV-WT titers in the lungs of challenged mice. **, Student’s *t* test (significance level less than 0.01); the results are expressed as the mean ± standard deviation. LOD, limit of detection.

**FIG 10:**
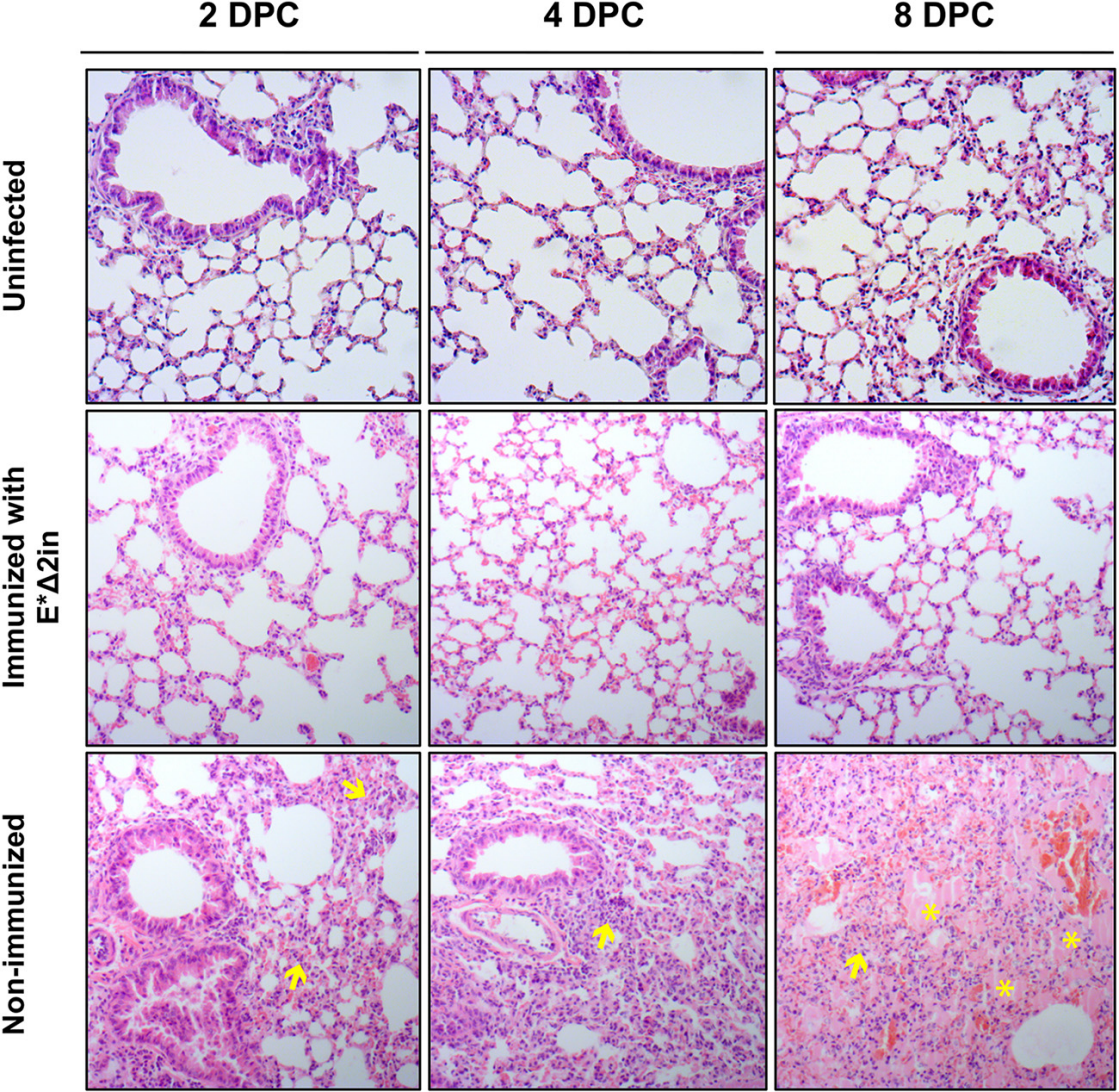
Histopathology of immunized and nonimmunized mice challenged with a high dose of MERS-CoV-WT. DPC, days postchallenge. The lungs of the mice immunized with the MERS-CoV-E*Δ2in mutant looked healthy throughout the experiment. In the lungs of nonimmunized mice, cellular infiltrates (yellow arrows) can be seen at 2 DPC, with highly evident edema (yellow asterisks) at 8 DPC.

No weight loss was observed in the immunized mice group, and all of them survived to challenge, demonstrating that immunization with the E*Δ2in mutant conferred protection even against a higher dose of MERS-CoV-WT (Fig. 9A). Compared to nonimmunized mice, the replication and transcription levels of MERS-CoV-WT were reduced throughout the course of the experiment in the lungs of the immunized mice, being undetectable at 8 dpc (Fig. 9B). No virus was detected in the lungs of the immunized mice at any time of sampling, in contrast to nonimmunized mice (Fig. 9C). Moreover, while the lungs of nonimmunized mice showed large infiltrates at 2 and 4 dpc, with appearance of edema at 8 dpc, the lungs of the immunized mice presented a healthy and functional aspect during the whole experiment (Fig. 10). All together, these results indicated that the protection elicited by the E*Δ2in mutant promoted a sterilizing immunity, as no histopathological damage or viable virus was observed in the lungs of the immunized mice.

### Stability of the attenuation of the E*Δ2in mutant.

**FIG 11:**
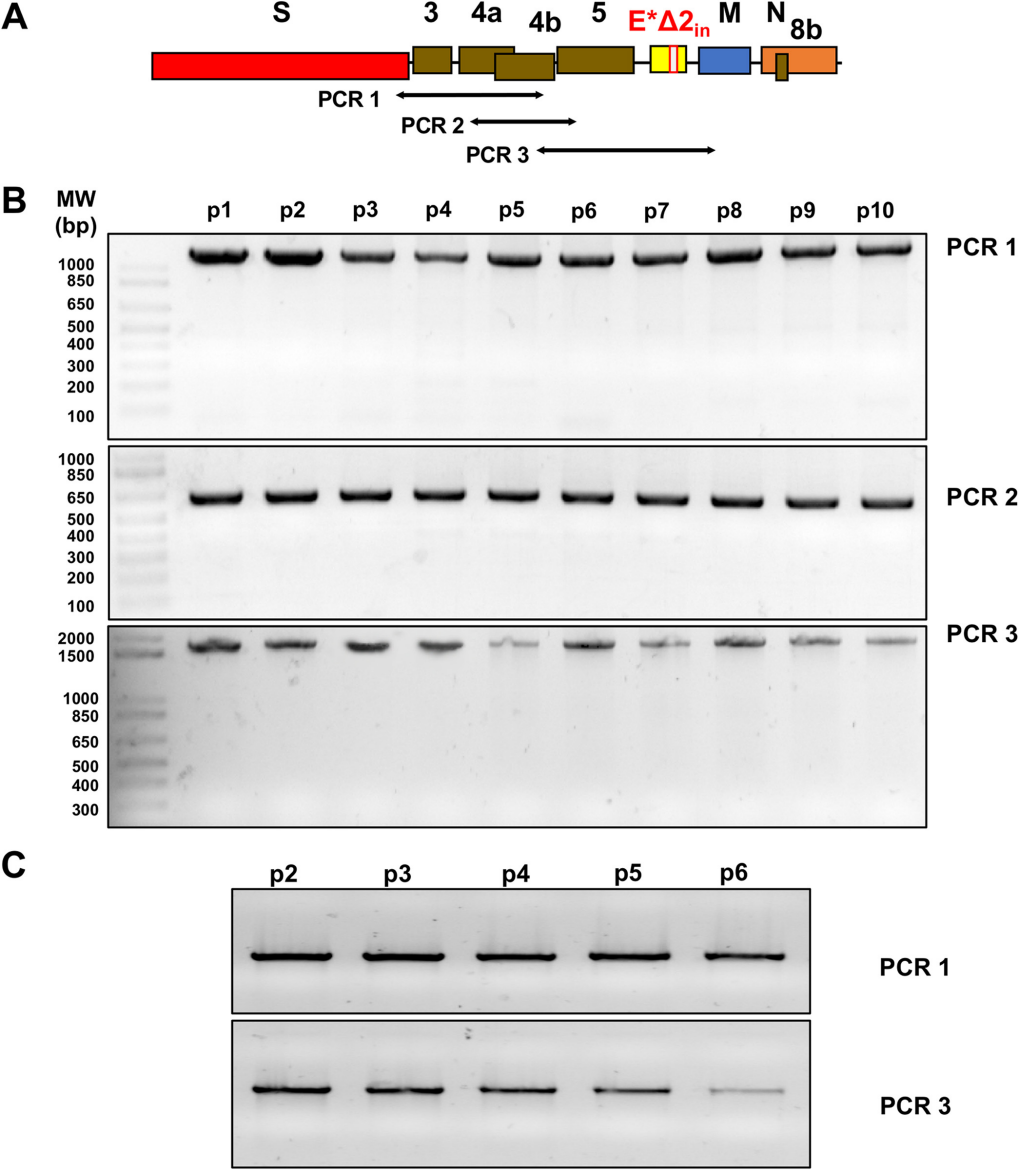
Stability of MERS-CoV-E*Δ2in mutant in Huh-7 and MRC-5 cells. (A) Amplified sequenced regions of the 3′ end of the genome. (B) Agarose gel electrophoresis of the PCR products of the RNAs from passages 1 to 10 in Huh-7 cells. (C) Agarose gel electrophoresis of the PCR products of the RNAs from passages 2 to 6 in MRC-5 cells.

Biosafety is one of the main concerns associated with live-attenuated vaccines. To determine the stability and the possibility of reversion to virulence, the E*Δ2in mutant and MERS-CoV-WT were passaged 10 times and 6 times in Huh-7 and MRC-5 cells, respectively. Then, the sequence of the final passage was compared with that of passage 1 virus (Fig. 11). It was observed that the mutation introduced in the E gene of the E*Δ2in mutant was stable after 10 and 6 passages in Huh-7 and MRC-5 cells, respectively.

**FIG 12:**
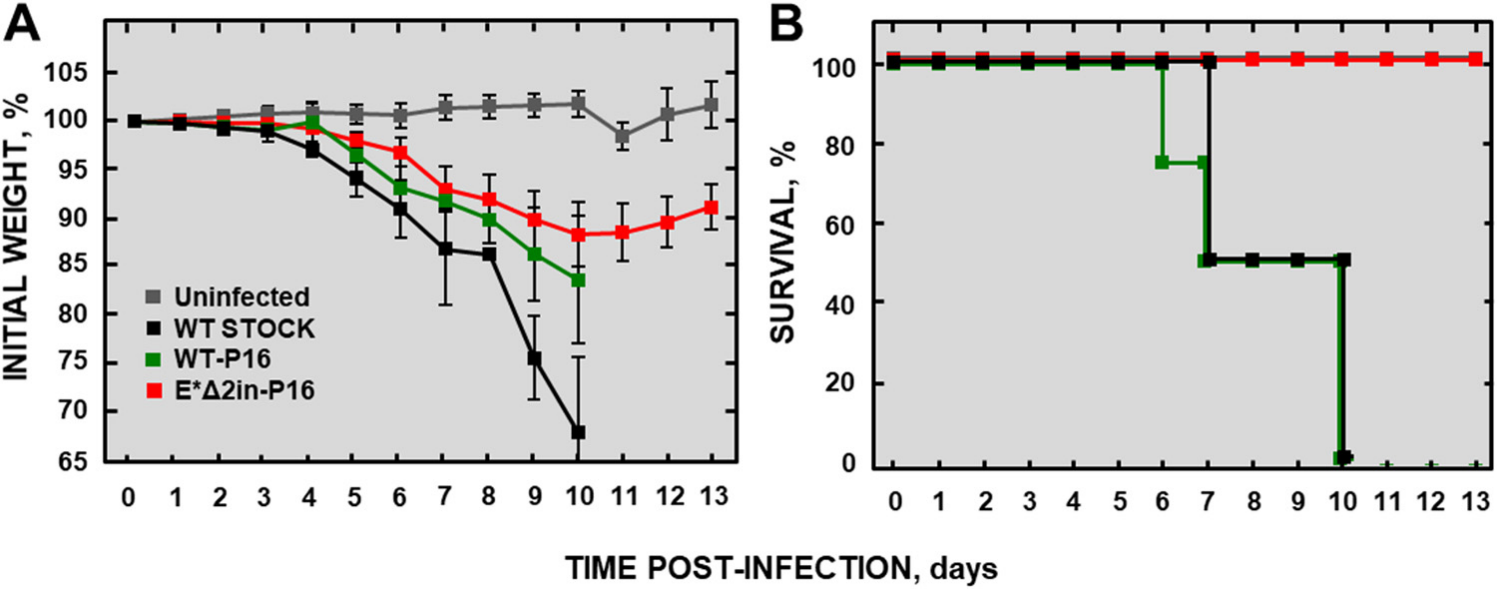
Evaluation of the attenuation of MERS-CoV-E*Δ2in in K18-hDPP4 mice after 16 passages in Huh-7 cells. (A) Weight loss of the infected mice. (B) Survival. Differences in weight loss are represented as the mean ± standard error of the mean. Stock WT, reference MERS-CoV-WT that has not been passaged in cell cultures. MERS-CoV-WT-P16, virulent virus passaged 16 times in cell cultures. MERS-CoV-E*Δ2in-P16, attenuated mutant passaged 16 times in cell cultures.

The E*Δ2in mutant and MERS-CoV-WT were then passaged six additional times in Huh-7 cells. The potential attenuation of the E*Δ2in virus obtained after 16 passages (E*Δ2in-P16) was evaluated *in vivo* (Fig. 12). The MERS-CoV-WT and the MERS-CoV-WT passaged 16 times (WT-P16) were used as controls. Both MERS-CoV-WT- and MERS-CoV-WT-P16-infected mice rapidly lost weight and died at 10 dpi. In contrast, all the mice inoculated with the E*Δ2in-P16 deletion mutant survived. Indeed, the sequences of the inoculated E*Δ2in-P16 deletion mutant and the E*Δ2in-P16 deletion mutant isolated from the lung of infected mice at 6 dpi were compared, and it was observed that both viruses retained the attenuated E gene sequence of the E*Δ2in mutant.

## DISCUSSION

Modern LAV are based on the study of the molecular mechanisms of pathology in viral infections, allowing the identification of viral genes specifically implicated in virulence. Through a reverse genetics system, these virulence factors can be deleted to obtain an attenuated virus of which we know its exact composition, pathogenicity, and stability, minimizing the risk of reversion to virulence (67). Currently, there are no approved vaccines against MERS-CoV infection for human use, while LAV can be designed and produced with a reverse genetics system like the one developed in our laboratory (81). In this work, a collection of MERS-CoV mutants have been generated by introducing small deletions within the C-terminal domain of the E protein (MERS-CoV-E* mutants). These deletions removed predicted functional sites. As a result, an attenuated vaccine candidate has been obtained that induced protection in a challenge with a lethal dose of MERS-CoV-WT. The characterization of the mutant led to the identification of a functional motif in the E protein, potentially related to MERS-CoV virulence.

From the five engineered deletion mutants of MERS-CoV E protein (MERS-CoV-E*), the E*Δ2 mutant was selected for further characterization. Apparently, three predicted linear motifs were deleted in the MERS-CoV-E*Δ1 mutant: the GSK3 motif, an Atg8 protein binding motif (Atg8-BM) and an FHA phosphopeptide ligand binding motif (FHA-BM) (82). Nevertheless, in the E protein sequence of the MERS-CoV-E*Δ1 mutant, the FHA-BM and the GSK3 motifs were recovered, but not the Atg8-BM one. Both the MERS-CoV-E*Δ1 mutant and MERS-CoV-WT were viable and virulent, and the unique difference between them was the absence of Atg8-BM, suggesting that this motif was not implicated in MERS-CoV virulence. All the mice infected with the MERS-CoV-E*Δ1 mutant lost weight and died just 1 day earlier than MERS-CoV-WT-infected mice (Fig. 5). This small difference may indicate the possible presence of an attenuating motif in E protein that, when removed, slightly increased virus virulence. In the MERS-CoV-E*Δ1 mutant, the only motif deleted was the Atg8-BM, whereas the attenuated MERS-CoV-E*Δ2in mutant lacks two known motifs: FHA-BM and Atg8-BM (Table 1 and also Table S1 in the supplemental material). It might be interesting to analyze which of these motifs is responsible for virus pathogenicity, an issue that will be addressed in future work.

**TABLE 1:** Relationship between viability and virulence of the MERS-CoV-E*Δ1, E*Δ2, and E*Δ2in mutants and the presence of different functional motifs of the MERS-CoV protein E^*a*^

Virus	Viability	Virulence	GSK3-PS	FHA-BM	Atg8-BM
E*Δ2	No	N.D.	No	No	No
E*Δ2in	Yes	No	Yes	No	No
E*Δ1	Yes	Yes	Yes	Yes	No
WT	Yes	Yes	Yes	Yes	Yes

a In this table, the results of the viability in cell cultures and the virulence *in vivo* of the mutants E*Δ1, E*Δ2, and E*Δ2in compared with the MERS-CoV-WT have been summarized. The columns indicate the presence (yes) or absence (no) of the three binding motifs in the protein E of each virus: FHA domain binding motif (FHA-BM), site of phosphorylation by GSK3 (GSK3-PS), and binding motif to proteins of the Atg8 family (Atg8-BM). N.D., not determined.

In the MERS-CoV-E*Δ2 mutant, a GSK3 phosphorylation site (GSK3-PS), an FHA-BM motif, and an Atg8-BM motif were deleted. The MERS-CoV-E*Δ2 mutant was not viable in cell culture. However, the spontaneous insertion of five amino acids (SQMVK), introduced into the protein E gene, gave rise to a viable virus (MERS-CoV-E*Δ2in) in which the GSK3-PS, but not the FHA-BM or the Atg8-BM, was restored. These data suggest that the presence of the GSK3-PS motif could be necessary for either replication or release of the virus. In this work, it has been shown that the attenuation of the mutant was not due to a decrease in the levels of replication or growth in the lung, since the MERS-CoV-E*Δ2in mutant grew and replicated at the same level as the MERS-CoV-WT. Likewise, it was observed that the MERS-CoV-E*Δ2in mutant did not produce significant pulmonary pathology compared to that produced by the MERS-CoV-WT. When comparing the E protein sequences of the MERS-CoV-E*Δ1 and MERS-CoV-E*Δ2in mutants, it was observed that they differed by the presence in MERS-CoV-E*Δ1 (virulent) or absence in MERS-CoV-E*Δ2in (attenuated) of the FHA-BM motif (Table 1 and Table S1). Taken together, these results suggest that FHA-BM may be involved in the virulence of MERS-CoV and brain tropism, which is relevant in the K18-hDDP4 mouse model, as it develops brain disease upon MERS-CoV infection (79). FHA domains are the only signaling domains that specifically recognize phosphothreonine residues (pThr) (83–86). These protein-phosphoprotein interaction modules are present in a wide variety of prokaryotic and eukaryotic proteins (87) and participate in various cellular functions such as signal transduction, vesicular trafficking, and cell cycle control (84, 88–96).

CoV E protein appears to be involved in many processes of viral morphogenesis, such as assembly, induction of envelope curvature, excision, or release of virions (97). In the case of MERS-CoV, the absence of the E gene led to a virus that is competent in replication but deficient in propagation (81). The requirement of protein E for virion maturation and release is different for each CoV. For example, partial deletions introduced in the SARS-CoV E gene attenuate the virus, but it is still viable (70), maybe because accessory protein 3a can partially compensate for deficiencies of protein E (71). In fact, the complete deletion of the E gene in SARS-CoV produces a viable virus, too, although the virus titer is reduced between 20 and 200 times (98). In the prediction of functional motifs of the MERS-CoV protein E, different potential domains were identified and deleted in each of the constructed MERS-CoV-E* mutants, leading to viable or nonviable viruses. MERS-CoV-E*Δ3 and MERS-CoV-E*Δ4 mutants were not viable, and MERS-CoV-E*Δ5 showed very low titers. The phenotype of these three mutants was like that observed when the E gene of the murine hepatitis virus (MHV) was mutated. In MHV, the replacement of several positively charged amino acids throughout the C-terminal domain by alanine residues leads to the appearance of temperature-sensitive mutants and to deficiencies in morphogenesis and virion release (99). Since protein E is key to the spread of MERS-CoV, it is likely that the deletions introduced into MERS-CoV-E*Δ3, MERS-CoV-E*Δ4, and MERS-CoV-E*Δ5 mutants were altering the structure of the E protein, rendering nonfunctional viruses, or a virus with reduced tissue tropism in the case of MERS-CoV-E*Δ2in.

The engineered MERS-CoV-E*Δ2in mutant seems to be the basis of a promising vaccine candidate, since it is an attenuated virus that conferred full protection against a lethal challenge with virulent MERS-CoV. Its high levels of replication and growth in cell cultures make it ideal from the point of view of large-scale vaccine production. Likewise, the E protein of the E*Δ2in mutant was stable after 10 passages in Huh-7 cells and 6 passages in MRC-5 cells; furthermore, the resulting virus after 16 passages in Huh-7 cells was attenuated *in vivo*. However, since the MERS-CoV-E*Δ2in mutant is a live-attenuated vaccine candidate, it will be mandatory to address what would be the potential spread of this mutant to contacts, and how stable it is *in vivo*. Besides, it is still necessary to introduce additional safety measures in order to administer this vaccine to humans. The introduction of partial deletions into the SARS-CoV nsp1 nonstructural protein has been shown in our laboratory to attenuate the virus *in vivo* (70). Mutations in the nsp16 protein of MERS-CoV also lead to virus attenuation (100). Additionally, accessory genes of MERS-CoV, involved in the antagonism of the interferon-mediated antiviral response, could be deleted (101–108) to introduce an additional safeguard. Incorporation of these deletions into the MERS-CoV-E*Δ2in mutant would increase its safety by considerably reducing the probability of reversion to a virulent phenotype in a recombination event.

## MATERIALS AND METHODS

### Ethics statement.

Animal experimental protocols were approved by the Environmental Council of Madrid (permit number: PROEX 112/14) and the Ethical Committee of the Center for Animal Health Research (CISA-INIA) (permit numbers: CBS 2014/005 and CEEA 2014/004) in strict accordance with Spanish National Royal Decree (RD 53/2013) and international EU guidelines 2010/63/UE about protection of animals used for experimentation and other scientific purposes and Spanish national law 32/2007 about animal welfare. All work with infected animals was performed in a biosafety level 3+ (BSL3+) laboratory of the Center for Animal Health Research (CISA-INIA, Madrid, Spain). Infected mice were housed in a self-contained ventilated rack (Allentown, NJ).

### Plasmids and bacterial strains.

Bacterial artificial chromosome (BAC) pBeloBAC11 (109), kindly provided by H. Shizuya (California Institute of Technology, Pasadena, CA), was used to assemble recombinant MERS-CoV infectious cDNA clones. This plasmid is a low-copy-number plasmid (one to two copies per cell) based on the Escherichia coli F factor (110) that allows the stable maintenance of large DNA fragments in bacteria. E. coli DH10B (Gibco/BRL) cells were transformed by electroporation using a MicroPulser unit (Bio-Rad) according to the manufacturer’s instructions. BAC plasmid and recombinant BACs were isolated and purified using a large-construct kit (Qiagen), following the manufacturer’s specifications.

### Generation of recombinant MERS-CoV infectious clones.

The cDNA from the MERS-CoV strain EMC/2012 (GenBank accession number JX869059), assembled into the pBAC (pBAC-SA-FL) (81), was used as the basis to generate a collection of five mutants with partial deletions within the C-terminal domain of the E protein. Each mutant has a deletion of 9 to 11 amino acids. Deletion fragments were generated by overlapping mutagenic PCR. Primers used for mutant engineering are described in Table S2 in the supplemental material. First, a PCR was performed with the oligonucleotide SA27502VS and the corresponding reverse-sense (RS) oligonucleotide of each mutant (PCR 1), and another PCR with the oligonucleotide SA28319RS and the corresponding virus-sense (VS) oligonucleotide of each mutant (PCR 2). As a template for these two PCRs, a pBAC was used that included nucleotides 25841 to 30162 of the MERS-CoV EMC/2012 genome (pBAC-SA-F6) and that contains the ORF4a, ORF4b, ORF5, E, M, and N viral genes. Between 50 and 150 ng of each product from PCRs 1 and 2 was taken as the template for a third PCR. The products of PCRs 1 and 2 were amplified with the oligonucleotides SA27502VS and SA28319RS, generating an overlapping PCR product (PCR 3) that included the partial deletion of the E gene flanked by the KflI and Pfl23II restriction sites.

10.1128/mBio.00103-21.3 TABLE S2 Sequences of the oligonucleotides used for the overlapping PCR for the generation of the MERS-CoV-E* mutants. Download Table S2, DOCX file, 0.02 MB. Copyright © 2021 Gutiérrez-Álvarez et al. 2021 Gutiérrez-Álvarez et al. https://creativecommons.org/licenses/by/4.0/ This is an open-access article distributed under the terms of the Creative Commons Attribution 4.0 International license.

### Cells.

Human hepatocyte-derived carcinoma (Huh-7), baby hamster kidney (BHK-21), and pulmonary human fibroblast-derived (MRC-5) cells were kindly provided by R. Bartenschlager (University of Heidelberg, Germany), H. Laude (Unité de Virologie et Immunologie Moléculaires, INRA, France), and the ATCC (CCL-171), respectively. Cells were grown in Dulbecco’s modified Eagle’s medium (DMEM) with 25 mM HEPES and 4.5 g/liter glucose (BioWhittaker Lonza), supplemented with 4 mM glutamine, 1× nonessential amino acids (Sigma-Aldrich), and 10% (vol/vol) fetal bovine serum (FBS) (HyClone Thermo Scientific).

### Viruses.

Parental wild-type and recombinant viruses, rescued from infectious cDNA clones generated in a BAC, were grown and titrated on Huh-7 cells using closed flasks or sealed plastic bags, respectively. All the work was performed at the CNB-CSIC biosafety level 3 facility (Madrid, Spain) following the security guidelines and standard procedures.

### Recovery of recombinant rMERS-CoV-E* mutants from the cDNA clones.

BHK cells were grown to 95% confluence in 12.5-cm^2^ flasks and transfected with 6 μg of infectious cDNA clone and 18 μl of Lipofectamine 2000 (Invitrogen), according to the manufacturer’s specifications. Three independent cDNA clones of each mutant were transfected. At 6 h posttransfection (hpt), cells were trypsinized, added to confluent Huh-7 cell monolayers grown in 12.5-cm^2^ flasks, and incubated at 37°C for 72 h. Cell supernatants were harvested (p0) and passaged four times on fresh cells. At passage 4 (p4), viability, titer, and sequence of the mutants were analyzed. Selected mutants were cloned by three rounds of plaque purification following standard procedures (81).

### Plaque assay.

Infected Huh-7 cells were overlaid with DMEM supplemented with 4 mM glutamine, 1× nonessential amino acids, 2% FBS, and 0.16 mg/ml DEAE-dextran, containing 0.6% low-melting-point agarose. At 72 h postinfection, cells were fixed with 10% formaldehyde and stained with 0.1% (wt/vol) crystal violet in 20% methanol for plaque counts (81).

### Focus-forming immunofluorescence assay.

Huh-7 cells at 5 × 10^4^ per well were seeded in 96-well plates in 100 μl of medium 1 day prior to the immunofluorescence assay. Next day, cells were infected with 20 μl of undiluted or serially 10-fold-diluted virus. At 16 h postinfection, cells were fixed with 4% (wt/vol) paraformaldehyde for 40 min, washed, and permeabilized with chilled methanol at room temperature (R/T) for 20 min. Unspecific binding sites were blocked with 10% FBS in phosphate-buffered saline (PBS) for 1 h at R/T. Then, cells were incubated for 90 min at R/T with polyclonal antibody rabbit anti-N-MERS (BioGenes, Germany). Secondary monoclonal antibody goat anti-rabbit conjugated with Alexa 488 (Invitrogen) was incubated for 45 min to detect and count MERS-CoV infectious foci.

### Growth kinetics.

Subconfluent monolayers (>90% confluence) of Huh-7 cells in 12.5-cm^2^ flasks were infected at a multiplicity of infection (MOI) of 0.001 with the indicated viruses. Culture supernatants were collected at 0, 24, 48, and 72 h postinfection (hpi), and virus titers were determined as described above.

### Mice.

MERS-CoV-susceptible transgenic mice (K18-hDPP4) were kindly provided by Paul McCray (University of Iowa, USA) (79). A colony was established in the CNB-CSIC animal care facility (Madrid, Spain). For infection experiments, female K18-hDPP4 mice at 16 to 24 weeks of age were anesthetized with isoflurane and intranasally inoculated. All work with infected animals was performed in a biosafety level 3+ facility by workers wearing personal protection equipment (3M).

### Virus infection and growth in mice.

K18-hDPP4 transgenic mice (79) were intranasally inoculated with 50 μl of DMEM mixed with virus. The indicated virus at 5 × 10^3^ PFU per mouse was inoculated for attenuation experiments, and 5 × 10^3^ PFU and 1 × 10^5^ PFU of MERS-CoV-WT (recombinant MERS-CoV reproducing the EMC/2012 strain engineered in the CNB-CSIC laboratory) (81) were used for the low- and high-dose challenge, respectively. Weight loss and mortality were evaluated daily. To determine viral titers, lungs and brains were homogenized in 2 ml of PBS containing 100 IU/ml penicillin, 0.1 mg/ml streptomycin, 50 μg/ml gentamicin, and 0.5 μg/ml amphotericin B (Fungizone), using a gentleMACS dissociator (Miltenyi Biotec, Inc.). Virus titrations were performed in Huh-7 cells as described above. Viral titers were expressed as PFU counts per gram of tissue.

### Extraction and analysis of viral RNA.

RNA from infected cells or homogenized mouse lungs was collected and purified using the RNeasy kit (Qiagen). Total cDNA was synthesized using a high-capacity cDNA reverse transcription kit (ThermoFisher Scientific) with random hexamers and 150 ng of purified RNA in a final volume of 30 μl. cDNA products were subsequently subjected to PCR for sequencing using Vent polymerase (New England Biolabs). Only cDNA products from mouse lungs were analyzed by real-time qPCR for viral RNA synthesis quantification. MERS-CoV genomic RNA (gRNA) (forward primer 5′-GCACATCTGTGGTTCTCCTCTCT-3′, reverse primer 5′-AAGCCCAGGCCCTACTATTAGC-3′, and minor groove binder (MGB) probe 5′-TGCTCCAACAGTTACAC-3′) and MERS-CoV subgenomic RNA (sgmRNA) N (forward primer 5′-CTTCCCCTCGTTCTCTTGCA-3′, reverse primer 5′-TCATTGTTATCGGCAAAGGAAA-3′, and MGB probe 5′-CTTTGATTTTAACGAATCTC-3′) custom assays were designed for this analysis; forward and reverse primers were purchased from Sigma-Aldrich, and MGB probes from Eurofins Genomics. Data were acquired with a 7500 real-time PCR system (Applied Biosystems) and analyzed with ABI Prism 7500 software, version 2.0.5. The relative quantifications were performed using the cycle threshold (2^−ΔΔ^*^CT^*) method (111). To normalize differences in RNA sampling, the expression of mouse 18S rRNA was analyzed using a specific TaqMan gene expression assay (Mm03928990_g1; ThermoFisher Scientific).

### Histopathology.

Mice were sacrificed at the indicated day postinfection or postchallenge. The left lung of infected mice was fixed in 10% zinc formalin for 24 h at 4°C and paraffin embedded. Serial longitudinal 5-μm sections were stained with hematoxylin and eosin (H&E) by the Histology Service at CNB-CSIC (Madrid, Spain) and subjected to histopathological examination with a Zeiss Axiophot fluorescence microscope. Samples were obtained using a systematic uniform random procedure, consisting in serial parallel slices made at a constant thickness interval of 50 μm. Histopathology analysis was conducted in a blind manner by acquiring images of 50 random microscopy fields from around 40 nonadjacent sections for each of the three independent mice analyzed per treatment group.

### Stability of mutants.

Stability of the selected MERS-CoV-E*Δ2in mutant was analyzed in Huh-7 and MRC-5 cells. Cells were seeded in 12.5-cm^2^ flasks and infected with the virus. Every 24 h, a third of the supernatant was passaged to a new 12.5-cm^2^ flask. After each passage, the remaining supernatant was conserved at −80°C, and the cells were lysed to extract the RNA as described above. The 3′-end third of the MERS-CoV genome (S, ORF3, ORF4a, ORF4b, ORF5, E, M, and N) was amplified by PCR, and analyzed by agarose gel electrophoresis and sequencing.

### Statistical analysis.

Two-tailed, unpaired Student’s t tests were used to analyze the differences in mean values between groups. All results were expressed as means ± standard deviations, except weight losses, which were expressed as means ± standard errors of the means; *P* values <0.05 were considered significant.
