# Supplementary material for: Genetically Engineered Live-Attenuated Middle East Respiratory Syndrome Coronavirus Viruses Confer Full Protection against Lethal Infection
Source: mBio. 2021 Mar 2;12(2):e00103-21. doi: 10.1128/mBio.00103-21 (PMC8092200; doi:10.1128/mBio.00103-21)
Supplement: TABLE S1 [file mBio.00103-21-st001.docx]

**TABLE S1. Prediction of functional motifs of MERS-CoV E protein.**

| Functional Class | Motif | Pattern | Sequence | Amino  acids | Deleted in mutants |
| --- | --- | --- | --- | --- | --- |
| GSK3 phosphorylation site | MOD_GSK3_1 | xxx([ST])xxx[ST] | QCMTGFNT | 42-49 | Δ2 |
| Atg8 protein family ligands | LIG_LIR_GEN_1 | [EDST]x[0,2][WFY]xxx[ILV] | TGFNTL | 45-50 | Δ1, Δ2, Δ2in |
| FHA phosphopeptide ligands | LIG_FHA_1 | xx(T)xx[ILV]x | FNTLLVQ | 47-53 | Δ2, Δ2in, (*Δ1) |
| SH2 ligands | LIG_SH2_STAT5 | (Y)[VLTFIC]xx | YLYN | 57-60 | Δ3 |
| NEK2 phosphorylation site | MOD_NEK2_1 | [FLM][^P][^P]([ST])[^DEP][^DE] | LYNTRG | 58-63 | Δ3 |
| SH2 ligands | LIG_SH2_STAT5 | (Y)[VLTFIC]xx | YVKF | 66-69 | Δ4 |
| Tyrosine-based localization signal | TRG_ENDOCYTIC_2 | Yxx[LMVIF] | YVKF | 66-69 | Δ4 |
| NEK2 phosphorylation site | MOD_NEK2_1 | [FLM][^P][^P]([ST])[^DEP][^DE] | FQDSKP | 69-74 | Δ4, Δ5 |
| SH3 ligands | LIG_SH3_3 | xxx[PV]xxP | DSKPPLP | 71-78 | Δ4, Δ5 |
| SH3 ligands | LIG_SH3_3 | xxx[PV]xxP | SKPPLPP | 72-78 | Δ4, Δ5 |
| WW domain ligands | LIG_WW_2 | PPLP | PPLP | 74-77 | Δ4, Δ5 |
| MYND domain binding motifs | LIG_MYND_1 | PxLxP | PPLPP | 74-78 | Δ4, ∆5 |
| PDZ ligands | LIG_PDZ_Class_3 | xxx[DE]x[ACVILF]$ | PPDEWV | 77-82 | Δ5 |
|  | | | | | |

(*Δ1): Deletion of the FHA domain binding motif (FHA phosphopeptide ligands) from the E*Δ1 mutant results in the formation of a new binding motif to FHA domains in the resulting sequence; therefore, this motif is not deleted in the E*Δ1 mutant. $: end of protein sequence. x: any amino acid. [ ]: any amino acids from those included in the square brackets. [^]: any amino acids except those included in the square brackets. ( ): important residue for motif functionality. x[0,2]: any 0, 1 or 2 amino acids. For more details, see The Eukaryotic Linear Motif resource for Functional Sites in Proteins (<http://elm.eu.org/)>.
