# Supplementary figures and images for: Genetically Engineered Live-Attenuated Middle East Respiratory Syndrome Coronavirus Viruses Confer Full Protection against Lethal Infection
Source: mBio. 2021 Mar 2;12(2):e00103-21. doi: 10.1128/mBio.00103-21 (PMC8092200; doi:10.1128/mBio.00103-21)

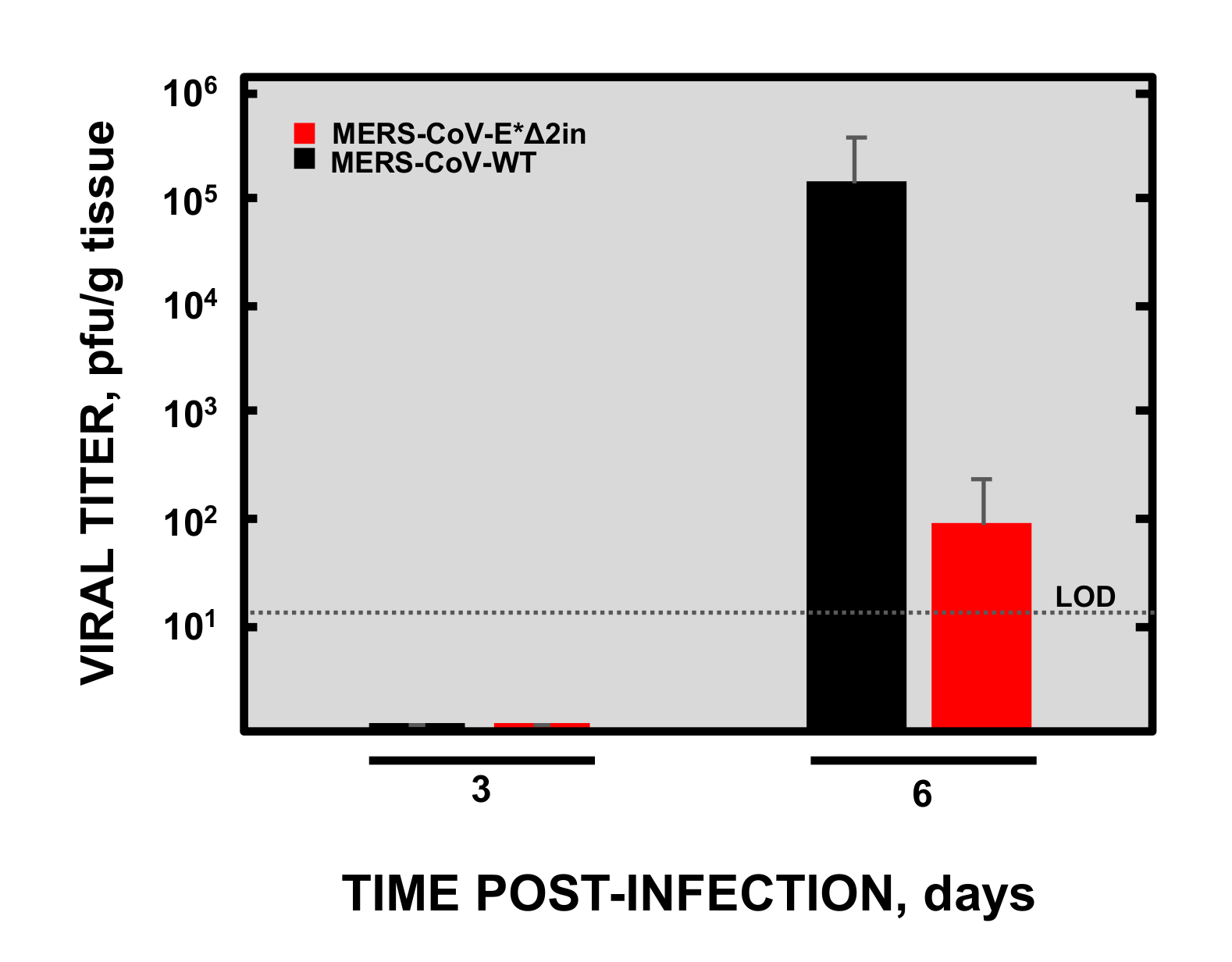

Supplement: FIG S1 [file mBio.00103-21-sf001.tif]
