# Supplementary material for: Genetically Engineered Live-Attenuated Middle East Respiratory Syndrome Coronavirus Viruses Confer Full Protection against Lethal Infection
Source: mBio. 2021 Mar 2;12(2):e00103-21. doi: 10.1128/mBio.00103-21 (PMC8092200; doi:10.1128/mBio.00103-21)
Supplement: TABLE S2 [file mBio.00103-21-st002.docx]

**TABLE S2. Sequences of the oligonucleotides used for the overlapping PCR for the generation of the MERS-CoV-E* mutants.**

| Oligo pair | Name | Sequence (5'->3') |
| --- | --- | --- |
| PCR 3 | **SA27502VS** | GCTTATCGTTTAAGCAGCTC |
|  | **SA28319RS** | TCTGTCGTAGTCACAAGCAC |
| E*Δ1 | **VS Δ1 - 2254-2283** | GCTTTCCTTACGGCTACTAATACCCTGTTAGTTCAGC |
|  | **RS Δ1 - 2254-2283** | GCTGAACTAACAGGGTATTAGTAGCCGTAAGGAAAGC |
| E*Δ2 | **VS Δ2 - 2281-2307** | GTGCAATGTATGACAGGCTTATACTTGTATAATACTGGACG |
|  | **RS Δ2 - 2281-2307** | CGTCCAGTATTATACAAGTATAAGCCTGTCATACATTGCAC |
| E*Δ3 | **VS Δ3 - 2305-2331** | ACCCTGTTAGTTCAGCCCTCAGTCTATGTAAAATTCCAGG |
|  | **RS Δ3 - 2305-2331** | CCTGGAATTTTACATAGACTGAGGGCTGAACTAACAGGGT |
| E*Δ4 | **VS Δ4 - 2338-2370** | CTTGTATAATACTGGACGTTCAGTCCCACCTGACGAGTGGGTT |
|  | **RS Δ4 - 2338-2370** | AACCCACTCGTCAGGTGGGACTGAACGTCCAGTATTATACAAG |
| E*Δ5 | **VS Δ5 - 2362-2388** | CTATGTAAAATTCCAGGATAGTAAATAACGAACTCCTTCATAATG |
|  | **RS Δ5 - 2362-2388** | CATTATGAAGGAGTTCGTTATTTACTATCCTGGAATTTTACATAG |
| *Tm: melting temperature. VS: Virus sense. RS: Reverse sense.* | | |
